# Clinical observation of Dan-Hong Hua-Yu oral solution in treating retinal vein occlusion

**DOI:** 10.1097/MD.0000000000020173

**Published:** 2020-05-22

**Authors:** Tie-Jun Li, Yu-Chen Sun, Qiu-Yan Ma, Yan Wu, Chao Yang, Nan Zhang, Yue Yang, Ying-Xin Yang

**Affiliations:** a Beijing Hospital of Traditional Chinese Medicine, Capital Medical University; b Dongzhimen Hospital, Beijing University of Chinese Medicine, Beijing 100700, China.

**Keywords:** central retinal vein occlusion, randomized controlled trial, Traditional Chinese medicine

## Abstract

**Introduction:**

Retinal vein occlusion refers to diseases with decreased vision, dilated tortuous retinal veins visible on the fundus, and retinal hemorrhage, edema, and osmosis distributed along the vein. There is still no ideal intervention to treat central retinal vein occlusion. This study plan to observe the efficacy of Dan-Hong Hua-Yu oral solution in treating non-ischemic retinal vein occlusion, in order to provide new treatment ideas.

**Methods/Design:**

We plan to use random number table method, 64 cases of non-ischemic central retinal vein occlusion that meet the inclusion criteria will be randomly divided into a treatment group and a control group. The intervention group will be treated with Dan-Hong Hua-Yu oral solution according to the syndrome differentiation of Traditional Chinese medicine and the patient's fundus condition. Each group will take 4 weeks as a course of treatment and three consecutive courses of treatment without any interval during the course of treatment. Changes of visual acuity, fundus performance, and total clinical symptoms of patients before and after treatment will be observed.

**Discussion:**

This study will observe the efficacy of Dan-Hong Hua-Yu oral solution in the treatment of non-ischemic central retinal vein occlusion, with a view to providing new treatment ideas.

**Trial registration:**

ClinicalTrials.gov, ChiCTR2000030625, Registered on March 08, 2020.

## 1 Introduction

Retinal vein occlusion (RVO) refers to diseases with decreased vision, dilated tortuous retinal veins visible on the fundus, and retinal hemorrhage, edema, and osmosis distributed along the vein.^[1]^ According to the site of occlusion, it can be divided into central branch retinal vein occlusion (CRVO) and branch retinal vein occlusion (BRVO). CRVO can be divided into ischemic and non-ischemic.^[2,3]^ RVO is a common retinal vascular disease in ophthalmology, and it is one of the major blinding fundus diseases. Due to the slow speed of venous blood flow, its severity is not as severe as that of arterial occlusion, but the venous occlusion has a longer incubation period, slow onset, and long course of disease, and its condition is very stubborn.^[4,5]^ Macular edema is the main complication of this disease, and vision often fails to recover. And, if the non-ischemic CRVO is not treated properly or timely, it can easily be converted into ischemic RVO, causing severe visual impairment.

The pathogenic factors of CRVO are relatively complex, and the etiology and pathogenesis are still unclear. According to previous surveys and studies, gender, age, hypertension, diabetes, atherosclerosis, cholesterol, high homocysteine, abnormal blood coagulation, glaucoma, refractive error, and intraocular pressure are all caused by retinal vein occlusion.^[6]^Among them, RVO is closely related to systemic diseases such as hypertension, diabetes, atherosclerosis, and high cholesterol. The CRVO is mostly related to hypertension, arteriosclerosis, high blood viscosity, and high intraocular pressure.^[7]^ The incidence of RVO is closely related to age, and its incidence increases with age. The recent studies have shown that the average age of onset of retinal vein occlusion is 64.8 years, while the average age of cases observed in China is 57.08 years.^[8,9]^ According to statistics, the incidence of RVO in China is as high as 1.9%, and an estimated 160 million people worldwide suffer from RVO.^[10,11]^ In recent years, with the improvement of people's living standards, changes in dietary structure, increased work and stress, and the acceleration of the pace of modern life, the incidence of this disease has increased, and its age of onset is getting lower and lower.^[12]^

The pathogenesis of CRVO is not very clear. The current treatment of CRVO is not ideal. Modern medicine now recognizes the treatment methods mainly to remove the cause, local and systemic treatment. Current treatment methods include laser therapy, vitreous cavity drug injection, combined therapy, hyperbaric oxygen therapy, surgical therapy, and drug therapy. Most modern medical treatments are aimed at preventing and treating complications, but the role of vision preservation and improvement is still controversial.^[13]^ At present, the surgical treatment of modern medicine, such as optic disc radial incision, its therapeutic effect and safety need to be further determined.^[14,15]^ The Traditional Chinese medicine (TCM) combined with the local ophthalmology syndrome at the same time as the whole body syndrome differentiation makes its treatment more complete. Because TCM has certain advantages in the treatment of CRVO, this study observed statistical changes in visual acuity, bleeding absorption range, and improvement of TCM symptoms of Dan-Hong Hua-Yu oral solution in the treatment of non-ischemic CRVO patients. We aim to provide new therapeutic ideas for the treatment of non-ischemic CRVO.

## 2 Methods/Design

### 2.1 Study design and settings

**Figure 1:**
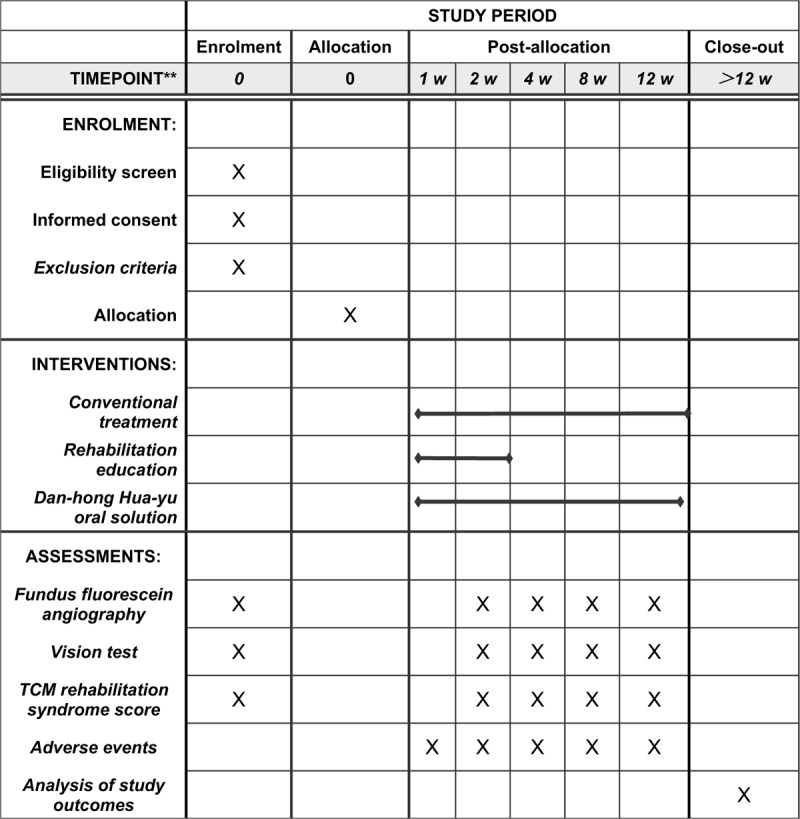
SPIRIT figure for the schedule of enrollment, interventions, and assessments.

This study will be a single-blinded, randomized controlled trial with two parallel groups. It will be conducted at the Beijing Hospital of Traditional Chinese Medicine, Capital Medical University. This protocol was written and based on Standard Protocol Items: Recommendations for Interventional Trials guidelines. If they agree, they will sign an informed consent form. Only those participants who read and agree to the protocol and who sign the informed consent form will take part in the study, following the schedule described in Figure 1.

### 2.2 Ethical aspects

This study will be approved by Beijing Hospital of Traditional Chinese Medicine Ethics Committee. It will be conducted in accordance with the protocol. The rules of confidentiality will be respected. The study protocol is funded through a protocol registry. The study is supported by the TCM evidence-based capacity building project.

### 2.3 Participants

All the cases in this study originated from the ophthalmology clinic and ward of Beijing Hospital of Traditional Chinese Medicine. Patients diagnosed with non-ischemic CRVO by clinical manifestations combined with fundus fluorescence angiography.

#### 2.3.1 Diagnostic criteria

The diagnostic criteria for western medicine will be formulated with reference to the “*Chinese Ophthalmology*” published by the People's Medical Publishing House in 2014 and the “*Traditional Chinese Medicine Disease Diagnostic Effectiveness Criteria*” issued by the State Administration of TCM in 2017. The details are as follows:

(1) Vision loss to varying degrees;
(2) (2) Fundus examination: normal or slightly blurred nipples, edema, dilated or tortuous veins, small or medium flaming retinas, and spot or sheet bleeding along the veins;
(3) Macular normal or mild edema;
(4) Fundus fluorescein angiography (FFA) shows prolonged retinal vein filling time, light capillary leakage, venous wall staining, no non-perfusion area, or neonatal blood vessels.

Non-ischemic CRVO is equivalent to the category of “blindness” in TCM. Therefore, the diagnostic criteria for TCM will refer to the standards of the Chinese medicine industry of the People's Republic of China for the “*Traditional Chinese Medicine Diagnosis and Efficacy Standards*”, the “Twelfth Five-Year Plan” Key Specialist Collaboration Group of the State Administration of TCM “*Diagnosis and Treatment of Blindness* (*Retinal Vein Occlusion*)” and “*Chinese Medicine Ophthalmology*” will be formulated.

#### 2.3.2 Inclusion criteria

This study will be conducted in China. We will enroll participants based on the following inclusion criteria:

(1) Those who meet the diagnosis of traditional Chinese and western medicine CRVO and have no other fundus lesions;
(2) Aged between 30 and 65 years;
(3) The onset of disease is within 1 month, and can be treated for 3 months;
(4) In the sign, after obtaining the consent of the patients and their families, they volunteered to participate in the research of this topic.

#### 2.3.3 Exclusion criteria

Patients will be excluded, if they meet the following criteria:

(1) Combined with other eye diseases that affect the determination of efficacy;
(2) Women during pregnancy or lactation;
(3) Patients who have taken hormonal drugs in the past 3 months or have been treated with this research protocol;
(4) People with severe heart, liver, and kidney dysfunction and mental disorders;
(5) Subjects participating in other clinical studies.

#### 2.3.4 Criteria for termination of trials

If the following happens, we will terminate the test: severe acute disease response, severe allergic reactions, and other adverse reactions; those who withdraw due to worsening or ineffective eye disease during treatment.

### 2.4 Interventions

The statistically significant minimum sample size for clinical trials requires no less than 30 patients in each group. For the selected cases that met the inclusion criteria and were screened out by the exclusion criteria, the subjects were randomly divided into the intervention group (32 cases) and the control group (32 cases) by random number method.

Control group: Patients in the control group will be treated with Fufang Xueshuantong Capsule (Manufacturer: Guangdong Zhongsheng Pharmaceutical Co., Ltd.; Specification: 0.5 g). Usage: oral, 1.5 g once, three times a day.

Intervention group: This group will be given the TCM prescription-Dan-Hong Hua-Yu oral solution (Guangzhou Baiyunshan Hutchison Huangpu Traditional Chinese Medicine Co., Ltd.) 20 mL/time, oral, three times a day. The composition of Dan-Hong Hua-Yu oral solution is: *Dansheng*, *Honghua*, *Chuanxiong*, *Taoren*, *Danggui*, *Caihu*, *Zhiqiao*.

Each group will take 4 weeks as a course of treatment and three consecutive courses of treatment without any interval during the course of treatment. At the same time, closely monitor the change of the condition in order to control the deterioration of the condition in time.

### 2.5 Outcome measures

#### 2.5.1 Primary outcome measures

*Fundus fluorescein angiography*. Inspection method: Tobiconamide eye drops in the conjunctival sac are fully dilated. Perform allergy tests on patients according to reagent requirements. If you are not allergic, push the sodium fluorescein stock solution into the vein and observe the patient's retinal vein filling with the fundus camera. Check before and after treatment.

#### 2.5.2 Secondary outcome measures

Observe the best corrected vision before and after treatment. Check with international standard eye chart. The visual acuity chart is 5 m away from the subject. During the inspection, the two eyes are performed separately. One eye is covered. Observe the changes of the fundus before and after treatment. Tobicalamide eye drops in the conjunctival sac were fully dilated, and the fundus camera was used to photograph the patient's fundus.

#### 2.5.3 Efficacy evaluation

Refer to “Guidelines for Clinical Research of New Chinese Medicine”. Efficacy was evaluated by the ratio of the difference between the points before and after treatment compared to the points before treatment. Syndrome treatment efficiency (%) = (total points before treatment total points after treatment)/total points before treatment × 100.

(1) Clinical control: clinical symptoms and signs disappeared or basically disappeared, and syndrome scores were reduced by ≥95%;
(2) Significant effect: clinical symptoms and signs were significantly improved, and syndrome scores were reduced by 70%;
(3) The signs and symptoms have improved, and the syndrome scores have decreased by ≥30%;
(4) Ineffective: the clinical symptoms and signs have not improved significantly, and the syndrome scores have decreased by <30%.

### 2.6 Sample size calculation

This study is a small sample clinical trial. According to statistical requirements, there are at least 30 cases in each group. Considering that the drop-out rate does not exceed 20%, a total of 64 cases will be collected, of which 32 will be in the intervention group and the other in the control group.

### 2.7 Randomization and blinding

The random method will use the closed envelope method for block randomization. Use the SAS software PROC PLAN procedure statement for random grouping. The subjects will be numbered 1 to 64 according to the order in which the patients were enrolled, and random numbers and assignments corresponding to the order of inclusion were obtained. The groups will be sealed in opaque envelopes, and the order of inclusion will be affixed to the surface of the envelopes. Randomization envelopes and distributions are performed by individuals who are not involved in treatment and evaluation.

### 2.8 Statistical analysis

According to the m-ITT principle, all randomized cases will be included. And there is at least one treatment and one post-baseline data. The missing data are not filled, and the actual observation values are used for statistical analysis. The measurement data are described by mean, standard deviation, median, and quartile. If the data meets the normal distribution, it is compared with the baseline value at the time of enrollment, and a paired *t* test is used. Differences in efficacy between the two groups will be compared using independent sample *t* tests. If the normal distribution is not satisfied, a non-parametric test is used. The count data is described using a composition ratio. If the normal distribution is satisfied, the changes before and after treatment in the two groups are tested by *X*^2^. If the normal distribution is not satisfied, a non-parametric test is used. The statistical software is SPSS 24.0, when *P* < .05, it showed that the difference is statistically significant.

### 2.9 Data management

**Figure 2:**
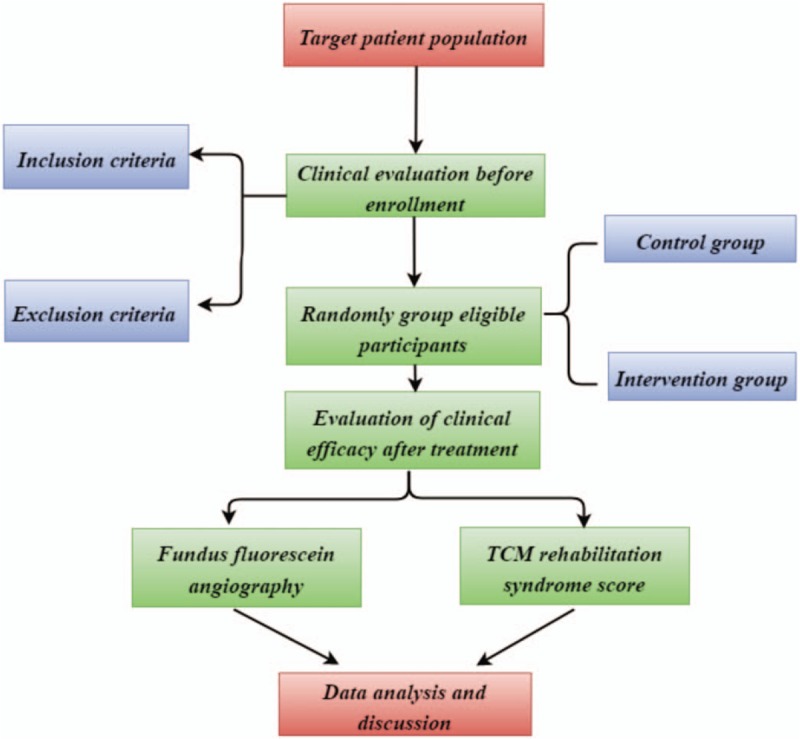
Study design flowchart.

Information obtained from the evaluation of each participant will be recorded on a paper print-out. The information will then be handwritten on a paper document case report form and entered into an Excel file for future statistical analyses. In accordance with the Personal Information Protection Act, the names of all participants will not be disclosed, and a unique identifier number given during the trial will be used to identify participants. All of the participants will be informed that the clinical data obtained in the trial will be stored in a computer and will be handled with confidentiality. The participants’ written consent will be stored by the principal investigator (Fig. 2).

## 3 Discussion

RVO is a more common fundus vascular disease. It is characterized by retinal blood stasis, tortuous dilation of the veins, retinal hemorrhage, and edema.^[16]^ RVO can be divided into central retinal vein occlusion and retinal vein branch occlusion. The occurrence of CRVO is closely related to systemic diseases, such as blood pressure, diabetes, atherosclerosis, and high cholesterol.^[17,18]^ But now the prevalence of chronic diseases such as hypertension, hyperlipidemia, diabetes, etc. are all showing a rapid increase in the prevalence of young age, and the age of the disease is getting younger and younger.^[19]^ In recent years, with the improvement of people's living standards, the change of dietary structure, the increase of work and living pressure, and the acceleration of the pace of life of modern people, the incidence of this disease has increased, and its occurrence has also become more and more young.^[20]^ The treatment of this disease is difficult, and some therapies are controversial. In theory, thrombosis is treated with anticoagulants, but the effect is not ideal. Many anticoagulants used in the past are no longer used. So far there is no special effective treatment. Generally, it can treat and prevent thrombosis according to the cause, such as lowering blood pressure and intraocular pressure, reducing blood viscosity, reducing thrombosis and tissue edema, and promoting bleeding absorption.^[21,22]^ From the perspective of clinical practice, there is still no ideal intervention for the treatment of CRVO. Modern medical treatment of CRVO is mainly aimed at the etiology treatment, prevention and treatment of complications.^[23]^ TCM has certain advantages in treating retinal vein occlusion.^[24]^ Dan-Hong Hua-Yu oral solution is a Chinese patent medicine. In the theory of TCM, it belongs to the blood treatment agent, which has the effects of promoting blood circulation and removing blood stasis, and promoting qi and collaterals. It is often used clinically for the treatment of blurred vision and central retinal vein occlusion caused by qi stagnation and blood stasis. This study will be to observe the efficacy of Dan-Hong Hua-Yu oral solution in treating non-ischemic CRVO, in order to provide new treatment ideas.

## Acknowledgments

The authors would like to thank all the trial participants. The authors are grateful for the support for this study: trial coordinating team, surgical staff, nurses, and research departments.

## Author contributions

TJL, YCS, QYM, and YW designed the study protocol and drafted the manuscript. CY and NZ reviewed the study protocol and drafted the manuscript. YY and YXY are responsible for the statistical design and analysis as trial statistician. All authors carefully read and approved the final version of the manuscript.
